# Prediction of mammalian virus cross-species transmission based on host proteins

**DOI:** 10.1128/spectrum.05368-22

**Published:** 2023-09-27

**Authors:** Zheng Zhang, Congyu Lu, Bocheng Mo, Kehan Bai, Xing-Yi Ge, Li Deng, Yousong Peng

**Affiliations:** 1 Bioinformatics Center, College of Biology, Hunan Provincial Key Laboratory of Medical Virology, Hunan University, Changsha, Hunan, China; 2 Hunan Engineering and Technology Research Center for Agricultural Big Data Analysis & Decision-making, College of Plant Protection, Hunan Agricultural University, Changsha, Hunan, China; 3 Hunan Juyoubiotech Co., Ltd, Changsha, Hunan, China; 4 Department of Internal Medicine-Neurology, The Third Hospital of Changsha, Changsha, Hunan, China; Shandong First Medical University, Jinan, Shandong, China

**Keywords:** mammalian virus, virus cross-species transmission, bioinformatics, zoonotic viruses

## Abstract

Most emerging viruses are spilled over from mammals. Understanding the mechanism of virus cross-species transmission and identifying zoonotic viruses before their emergence are critical for the prevention and control of newly emerging viruses. This study systematically investigated the host proteins associated with the cross-species transmission of mammalian viruses based on 1,271 pairs of virus-mammal interactions including 382 viruses from 33 viral families and 73 mammal species from 11 orders. Numerous host proteins were found to contribute to the cross-species transmission of mammalian viruses. Host proteins potentially contributing to virus cross-species transmission are specific to viral families, and few overlaps of such host proteins are observed in different viral families. Based on these host proteins, the random-forest (RF) models were built to predict the cross-species transmission potential of mammalian viruses. Moderate performance was obtained when using all viruses together. However, when modeling by viral family, the performance of the RF models varied much among viral families. In 13 viral families such as *Flaviviridae*, *Retroviridae,* and *Poxviridae*, the AUC of the RF model was greater than 0.8. Finally, the contribution of virus receptors to cross-species transmission was evaluated, and the virus receptor was found to have a minor effect in predicting the cross-species transmission of mammalian viruses. The study deepens our understanding of the mechanism of virus cross-species transmission and provides a framework for predicting the cross-species transmission of mammalian viruses.

**IMPORTANCE:**

Emerging viruses pose serious threats to humans. Understanding the mechanism of virus cross-species transmission and identifying zoonotic viruses before their emergence are critical for the prevention and control of emerging viruses. This study systematically identified host factors associated with cross-species transmission of mammalian viruses and further built machine-learning models for predicting cross-species transmission of the viruses based on host factors including virus receptors. The study not only deepens our understanding of the mechanism of virus cross-species transmission but also provides a framework for predicting the cross-species transmission of mammalian viruses based on host factors.

## INTRODUCTION

During the last 20 years, several global epidemic or pandemic diseases have been caused by emerging and re-emerging viruses, such as SARS-CoV (1), H1N1 virus (2), H7N9 virus (3), Ebola virus (4), and SARS-CoV-2 (5). The current COVID-19 pandemic, caused by SARS-CoV-2, has resulted in more than 767,972,961 human infections and 6,950,655 deaths globally as of 12 July 2023 (6). It is foreseeable that newly emerging viruses will continue to cause epidemics or pandemics in the future, posing a great threat to global public health. Most emerging human pathogens are spilled over from mammals. For example, both SARS-CoV and Ebola viruses were reported to originate from bats (7, 8). Understanding the mechanism of virus cross-species transmission and identifying zoonotic viruses before they emerge are critical for effectively preventing and controlling newly emerging viral pathogens.

Many factors influencing the cross-species transmission of mammalian viruses have been reported, which can be roughly classified into two categories. The first category comprises macro-level factors including ecological, social-economic, evolutionary, and biological factors that determine whether the virus can access the novel host (9). In a previous study conducted by Olival et al., they systematically investigated the host and viral traits that contributed to the spillover of mammalian viruses into humans (10). They found that the ratio of zoonotic viruses per mammal species was well predicted by the phylogenetic relatedness of the species to humans, the taxonomy of the species, and the extent of contact between the species and humans. Additionally, phylogenetic host breadth and other viral traits such as the transmission mode and whether the virus is enveloped or not were also significant predictors of the zoonotic potential of viruses. Valero-Rello and Sanjuán demonstrated that enveloped viruses have a higher likelihood of spillover than non-enveloped viruses, while other viral traits such as genome composition, structure, size, or the viral replication compartment play a less obvious role, by analyzing over 12,000 mammalian virus-host interactions (11). Furthermore, Grange et al. developed a risk ranking framework that integrated host, viral, and environmental risk factors using expert opinion and scientific evidence to estimate a risk score for wildlife-origin viruses (12).

The second category comprises molecular-level factors, including viral and host factors, which determine whether the virus can infect the host cell. In terms of viral factors, the viral receptor-binding protein and other viral proteins determine whether the virus can infect the host cells (13, 14). In terms of host factors, the virus receptor, which is responsible for viral entry into host cells, is reported to determine the host susceptibility to viruses to a large extent. For example, the ACE2 protein, which is the main receptor for SARS-CoV-2, has been used to assess the susceptibility of various species to the virus (15). In a previous study conducted by Cho et al., it was found that receptor similarity could be an excellent predictor for cross-species infection propensities of viruses (16). The antiviral genes that limit virus invasion such as the interferon also influence host susceptibility to viruses (17, 18). For example, the MX1 gene that encodes the myxovirus resistance protein A, an interferon-induced antiviral guanosine triphosphatase, has been reported to play a crucial role in human susceptibility to the H7N9 virus (19). Moreover, lots of host factors that are essential for viral replication, transcription, and other steps in the viral life cycle have been identified through protein-protein interaction analysis, using either experimental or computational methods. They are also important for the virus cross-species transmission (20 – 24). However, few studies have quantified the extent of host or viral factors in determining the cross-species transmission of viruses.

This study systematically investigated the host proteins associated with the cross-species transmission of mammalian viruses by viral family and further evaluated the effect of host proteins and viral receptor proteins in predicting the cross-species transmission of mammalian viruses. It deepens our understanding of the mechanism of virus cross-species transmission and provides a framework for predicting the cross-species transmission of mammalian viruses.

## MATERIALS AND METHODS

### Workflow of the study

**Fig 1:**
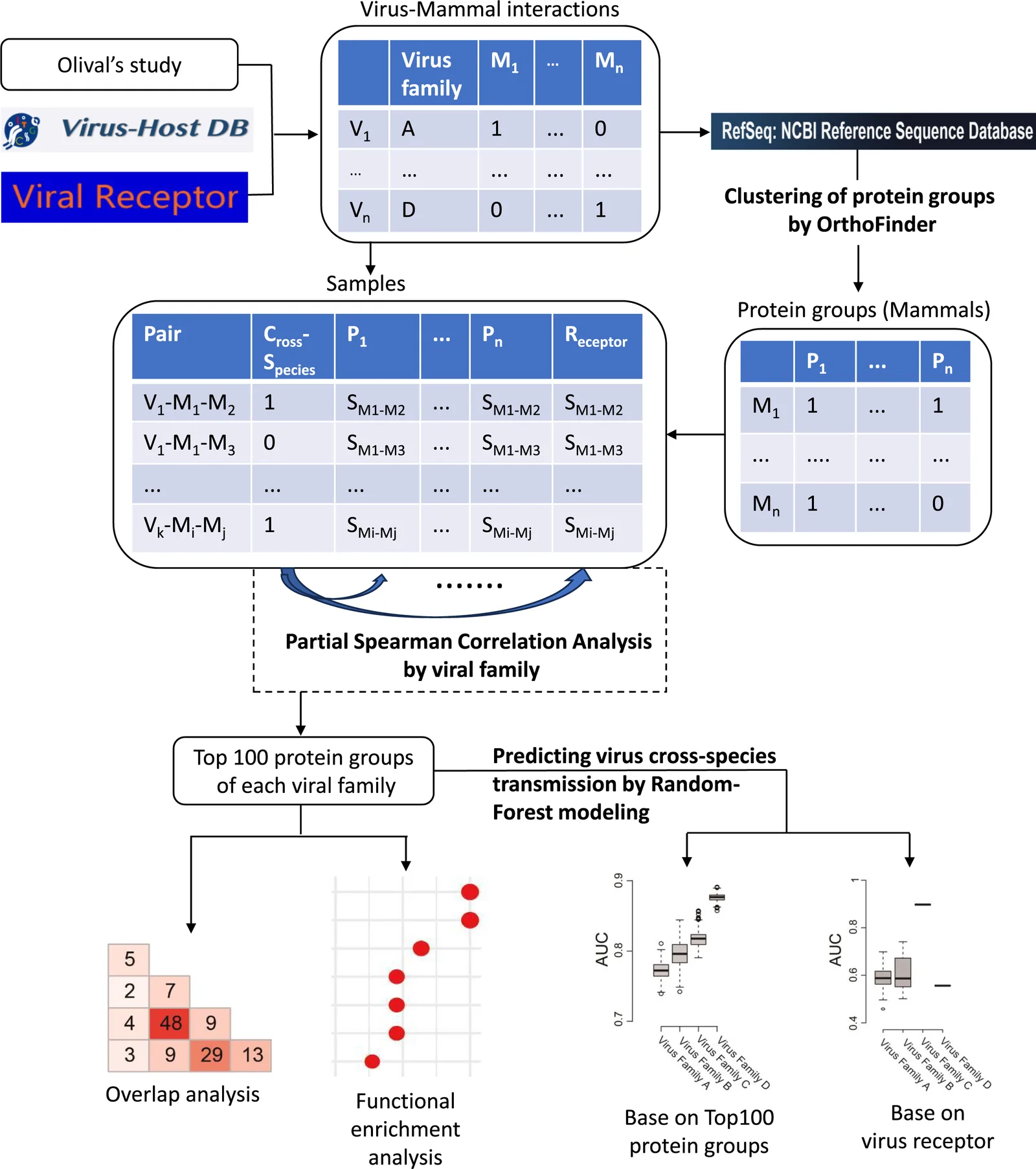
The workflow of the study. S_Mi-Mj_ refers to the sequence similarity between the proteins in M_i_ and M_j_.

The workflow of the study is shown in Fig. 1. First, positive and negative samples were generated based on the interactions between viruses and mammal species. Subsequently, the protein groups were generated, and their correlations to the cross-species transmission of mammalian viruses were analyzed by viral family. Next, the protein groups and the virus receptors were utilized to predict the viral cross-species transmission using machine-learning algorithms. The details of these processes are described in the following sections.

### Interactions between viruses and mammal species

The interactions between viruses and mammal species were derived from three sources: the Olival’s study, which curated 2,805 pairs of virus-mammal interactions from the literature (10), the Virus-Host database (25), and the ViralReceptor database (26). Only interactions with detailed virus taxonomy in the NCBI Taxonomy database and with the proteome of mammals available in the NCBI RefSeq database were retained. The viruses in the interactions were organized by viral species. A total of 1,271 pairs of virus-mammal interactions were obtained and used in the study, including 382 virus species from 33 viral families and 73 mammal species that were infected by two or more viruses (Table S1).

### Proteomes of mammal species

The proteome of mammal species, including humans, was obtained from the NCBI RefSeq database on 26 March 2021.

### Clustering of protein groups

Proteins in 73 mammal species were clustered by OrthoFinder (version 2.0) (27) with default parameter settings. A total of 38,300 protein groups were obtained, among which 16,943 protein groups that had members in no less than 50 mammal species were used for further analysis (Fig. S1). The protein sequences in each protein group were aligned by MAFFT (version 7.271) (28). The pairwise sequence similarities within each protein group were calculated based on the BLOSUM62 matrix.

### Correlations between cross-species transmission of mammalian viruses and protein groups

To evaluate the associations between the cross-species transmission of mammalian viruses and protein groups, the correlations between the protein sequence similarity within a protein group and the occurrence of virus cross-species transmission between mammals were calculated. The partial Spearman correlation coefficient (PSCC) was used to remove the influence of the phylogeny of mammal species on the correlations between viral cross-species transmission and protein groups, with cytochrome b as the covariable. The PSCC was calculated with the tool pingouin (version 0.5.0, available at https://pingouin-stats.org/index.html#) (29). The *P*-value of the PSCC was adjusted with the *q*-value method. Only the PSCCs with *q*-values <0.01 were retained for further analysis.

### Functional enrichment analysis

The gene ontology (GO) and Kyoto Encyclopedia of Genes and Genomes (KEGG) pathway enrichment analysis of human proteins were conducted with the functions of *enrichGO()* and *enrichKEGG()* in the packages “clusterProfiler” (version 4.8.1) (30) and org.Hs.eg.db (version 3.17.0) (31) in R (version 4.3.1) (32).

### Prediction of viral cross-species transmission using the random-forest algorithm

For each virus, a pair of mammal species both of which could be infected by the virus was considered a positive sample, while that with only one mammal species infected by the virus was considered a negative sample (Fig. 1). The sequence similarities within protein groups were used as features in the modeling. As not all protein groups cover all mammal species (Fig. S1), samples with more than 10% missing values were discarded in the modeling. The random-forest (RF) algorithm is an ensemble machine learning algorithm that uses multiple decision trees and can handle data with high variance and bias while reducing the risk of over-fitting by averaging multiple trees. Therefore, the RF algorithm was chosen to predict virus cross-species transmission between mammals. Due to the imbalanced nature of the data set, where the number of positive samples was much smaller than that of negative samples, the function of *BalanceRandomForest* in the package “imbalanced-learn” (version 0.8.0) (33) in Python (version 3.8.8) was used to conduct the RF modeling with the parameter of n_estimators set to be 100.

The leave-one-out test by mammal species was used to evaluate the performance of the RF model. In this approach, one mammal species was excluded from the data set each time, and the samples associated with the excluded mammal species were used as the testing data, while the remaining data were used as the training data. This process was repeated for each mammal species in the data set. The predictive performance of the RF model was measured by the area under the receiver operating characteristics curve (AUC).

To reduce the bias of virus-host interactions in the modeling, samples from three mammal species in each order were randomly selected for modeling, and this process was repeated 1,000 times.

### Prediction of mammalian virus cross-species transmission based on virus receptors

A total of 103 virus receptor proteins of 72 mammalian viruses were obtained from the ViralReceptor database (26). These receptor proteins belonged to the 72 protein groups mentioned above (Table S2). As shown in Fig. 1, when using the receptor proteins to predict the mammalian virus cross-species transmission, for a sample of V_k_-M_i_-M_j_, the protein groups containing the receptors of V_k_ were identified; then, the pairwise sequence similarities between proteins in M_i_ and those in M_j_ were calculated for each protein group, the average similarity was computed for each protein group, and the maximum average similarity was considered the similarity between virus receptors in M_i_ and M_j_ (S_Mi-Mj_); finally, S_Mi-Mj_ was used to predict whether the virus V_k_ had cross-species transmission between M_i_ and M_j_.

## RESULTS

### Interactions between viruses and mammal species

**Fig 2:**
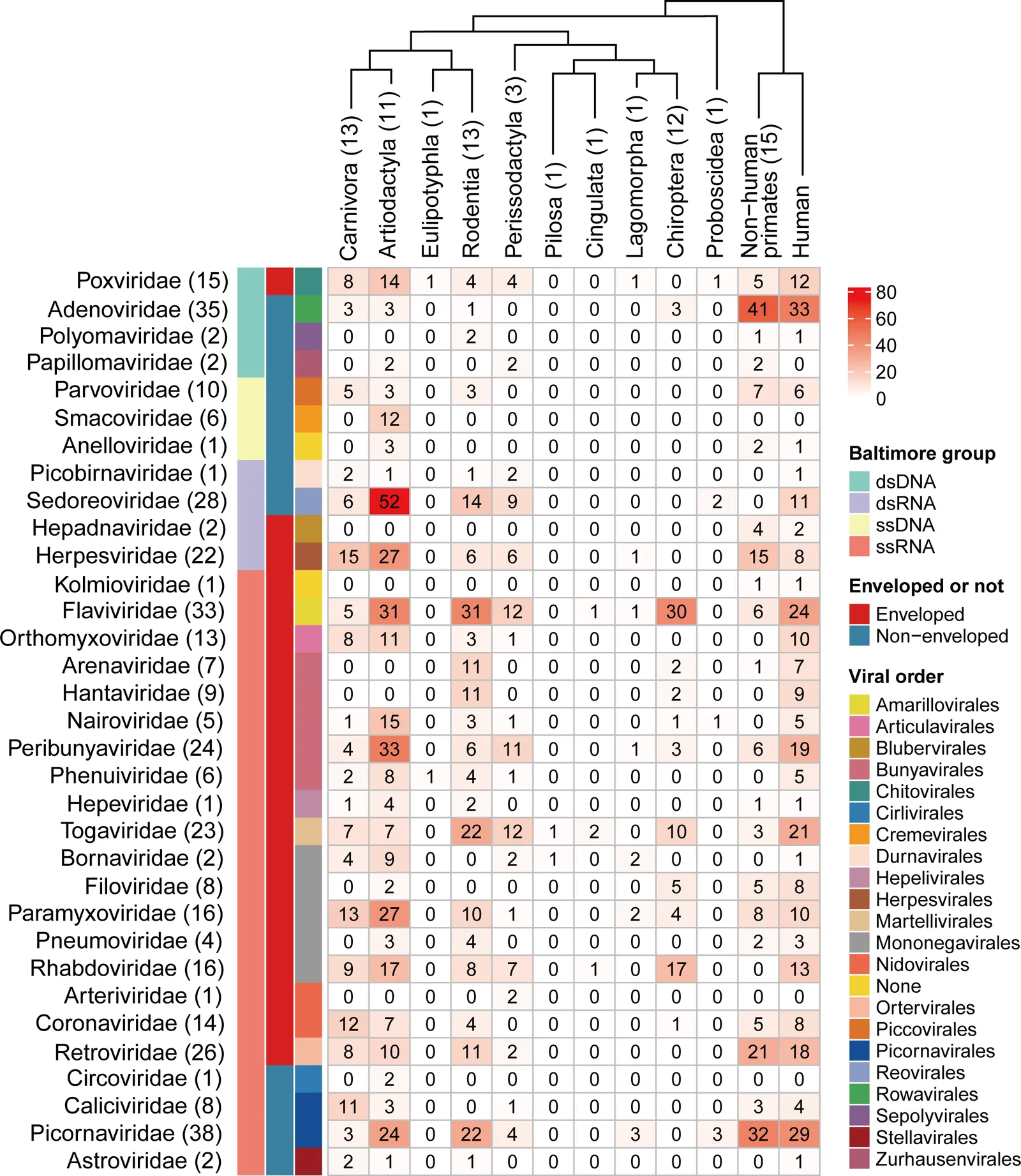
The virus-mammal interactions by viral family and host order. The figure was colored according to the number of interactions between the virus and the host. The viral family is shown on the left side of the figure. The numbers beside the viral family name refer to the number of viruses in the family used in the study. The Baltimore group, viral order, and enveloped or not of viral families are shown on the left side of the figure. The host order was shown at the top of the figure and was clustered based on cytochrome b. The numbers beside the host order refer to the number of mammal species in the order used in the study.

A total of 1,271 pairs of virus-mammal interactions were obtained, which included 382 viruses from 33 viral families and 73 mammal species from 11 orders (Table S1; Fig. 2). Most viral families belonged to the single-stranded RNA virus and enveloped viruses. The viral families of *Flaviviridae*, *Picornaviridae*, and *Sedoreoviridae* had higher number of interactions with mammals. For example, the Saint Louis encephalitis virus in the *Flaviviridae* family and the encephalomyocarditis virus in the *Picornaviridae* family infected 15 and 12 mammal species, respectively (Table S1). Regarding mammals, humans were the most infected by viruses (272 viruses), followed by the orders of *Artiodactyla*, *Primates* (excluding humans), and *Rodentia*. For example, the *Bos taurus* in the *Artiodactyla* was infected by 94 viruses from 20 viral families (Table S1).

### Correlations between cross-species transmission of mammalian viruses and protein groups

**Fig 3:**
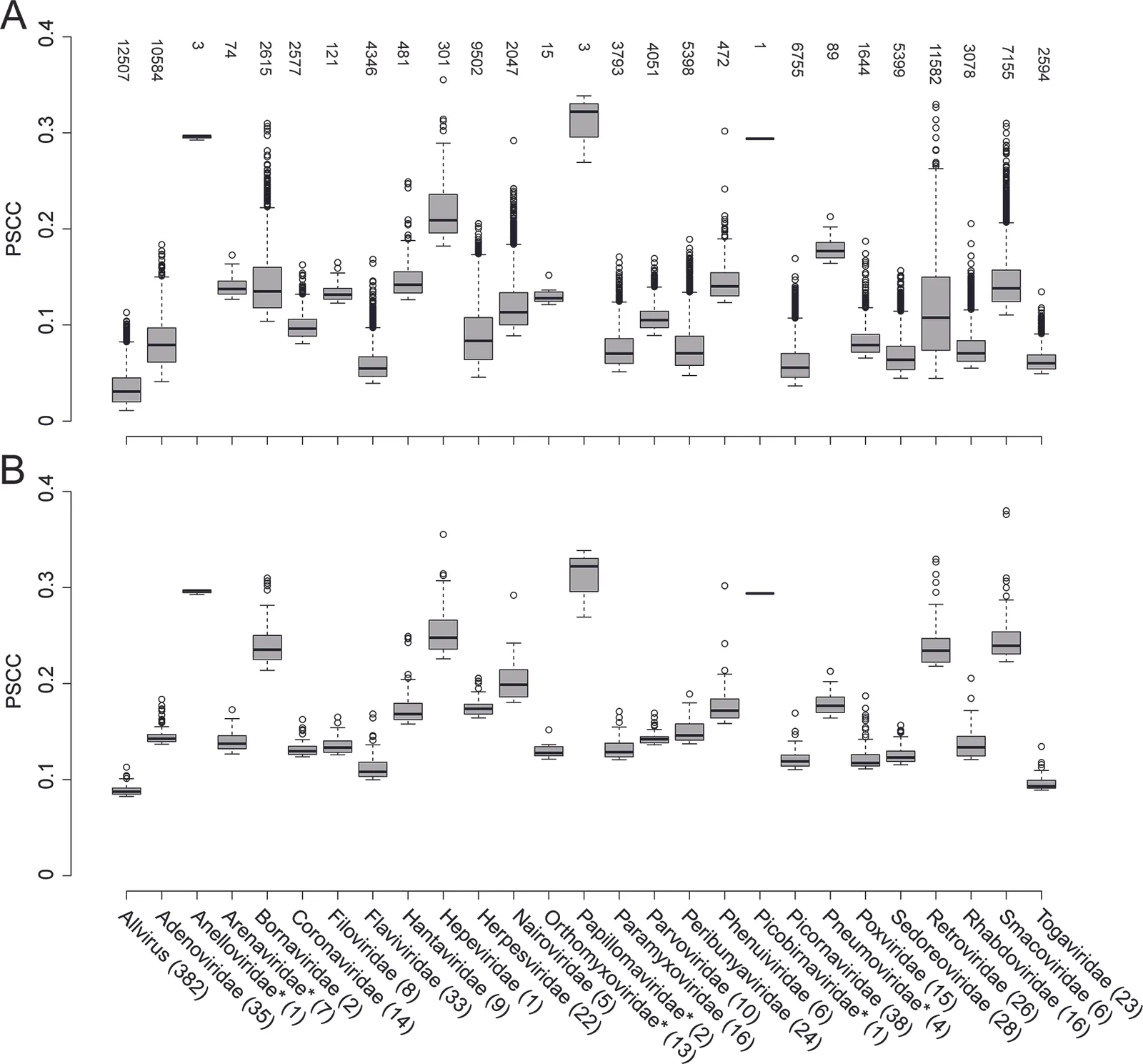
The distribution of PSCCs between protein groups and mammalian virus cross-species transmission for all viruses and each viral family. (**A**) The distribution of the absolute PSCCs of all protein groups. The numbers on top of the figure refer to the number of protein groups with significant correlations. (**B**) The distribution of PSCCs of the top 100 most correlated protein groups. “*” indicates the viral families with less than 100 protein groups that had significant correlations to the virus cross-species transmission. For clarity, seven viral families, including *Arteriviridae, Astroviridae, Caliciviridae, Circoviridae, Hepadnaviridae, Kolmioviridae, and Polyomavirida,* which had no protein groups associated with virus cross-species transmission, were not shown here.

A total of 16,943 protein groups that had members in more than 50 mammal species were used for further analysis (Fig. S1). The correlations between protein groups and virus cross-species transmission were analyzed (Fig. 3). When considering all viruses together, all protein groups showed low or no correlations with the mammalian virus cross-species transmission, with a median PSCC of 0.031. However, when analyzing the associations by viral family, increased correlations were observed between protein groups and virus cross-species transmission for most viral families (Fig. 3A). For example, the viral families of *Pneumoviridae* and *Coronaviridae* had a median PSCC of 0.177 and 0.096, respectively. When considering the top 100 protein groups that correlated the most with virus cross-species transmission, the median PSCCs were >0.15 for 12 viral families such as *Bornaviridae* and *Retroviridae* (Fig. 3B).

**Fig 4:**
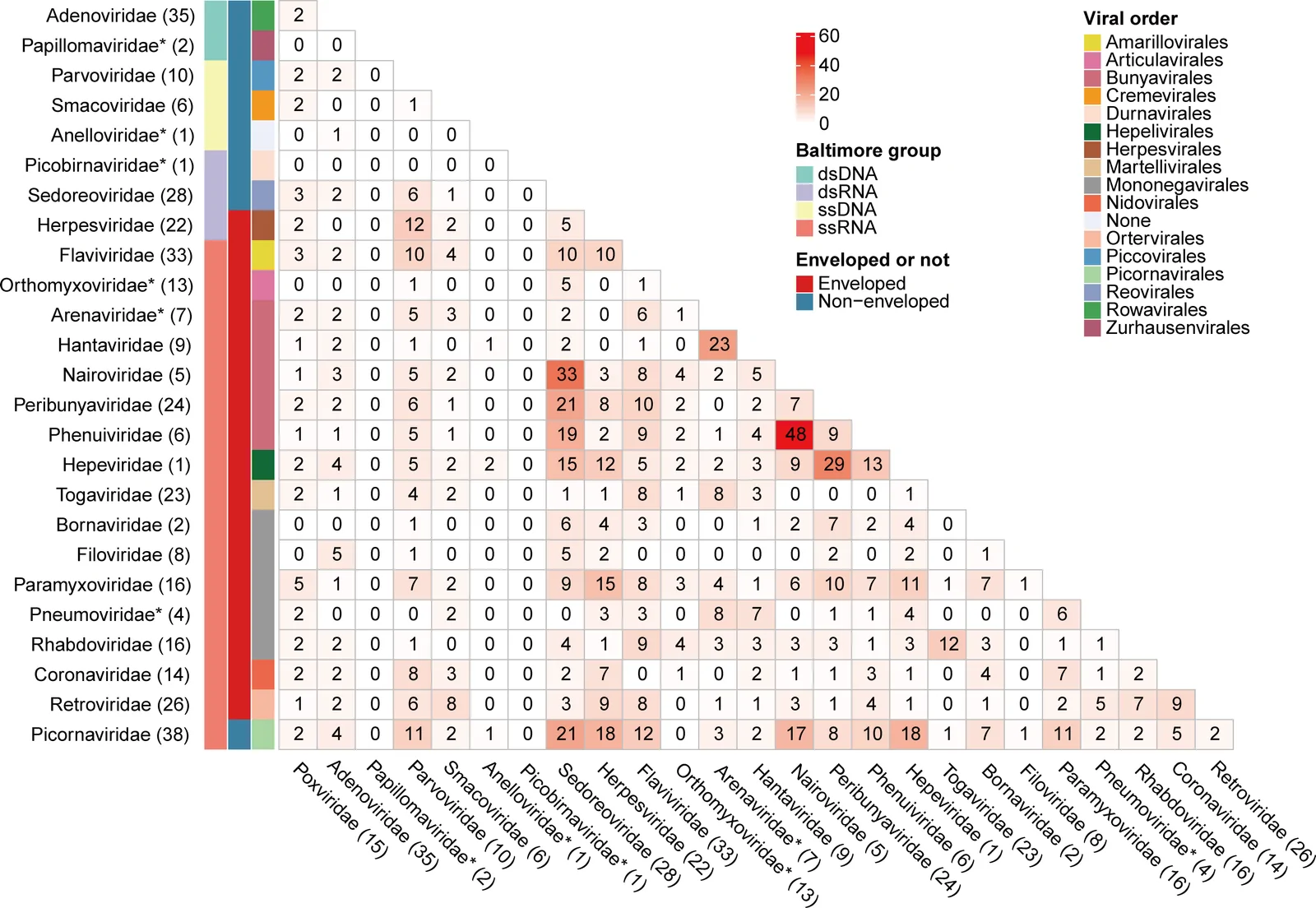
The overlap of the top 100 protein groups between different viral families. The Baltimore group, viral order, and enveloped or not of viral families were shown on the left side of the figure. “*” indicates the viral families with less than 100 protein groups that had significant correlations with virus cross-species transmission.

Then, the overlaps between the top 100 protein groups of different viral families were analyzed. As shown in Fig. 4, for most viral families, only a few protein groups were shared with other viral families. For example, the *Coronaviridae* had an average of two protein groups shared with other viral families. However, some viral families were observed to share a large ratio of protein groups with one or several other viral families. For example, the *Nairoviridae* shared 48 and 33 protein groups with *Phenuiviridae* and *Sedoreoviridae*, respectively (Fig. 4).

We also analyzed the frequency of protein groups among the top 100 protein groups of multiple virus families. Most protein groups (74%) were only observed in one viral family. However, there were 21 protein groups observed in five or more viral families (Table S3). For example, the protein group with the human protein Transmembrane protein 250, which was present in most (eight) viral families, has been reported to promote virus proliferation.

### Functional analysis of the top 100 protein groups associated with virus cross-species transmission in several viral families

**Fig 5:**
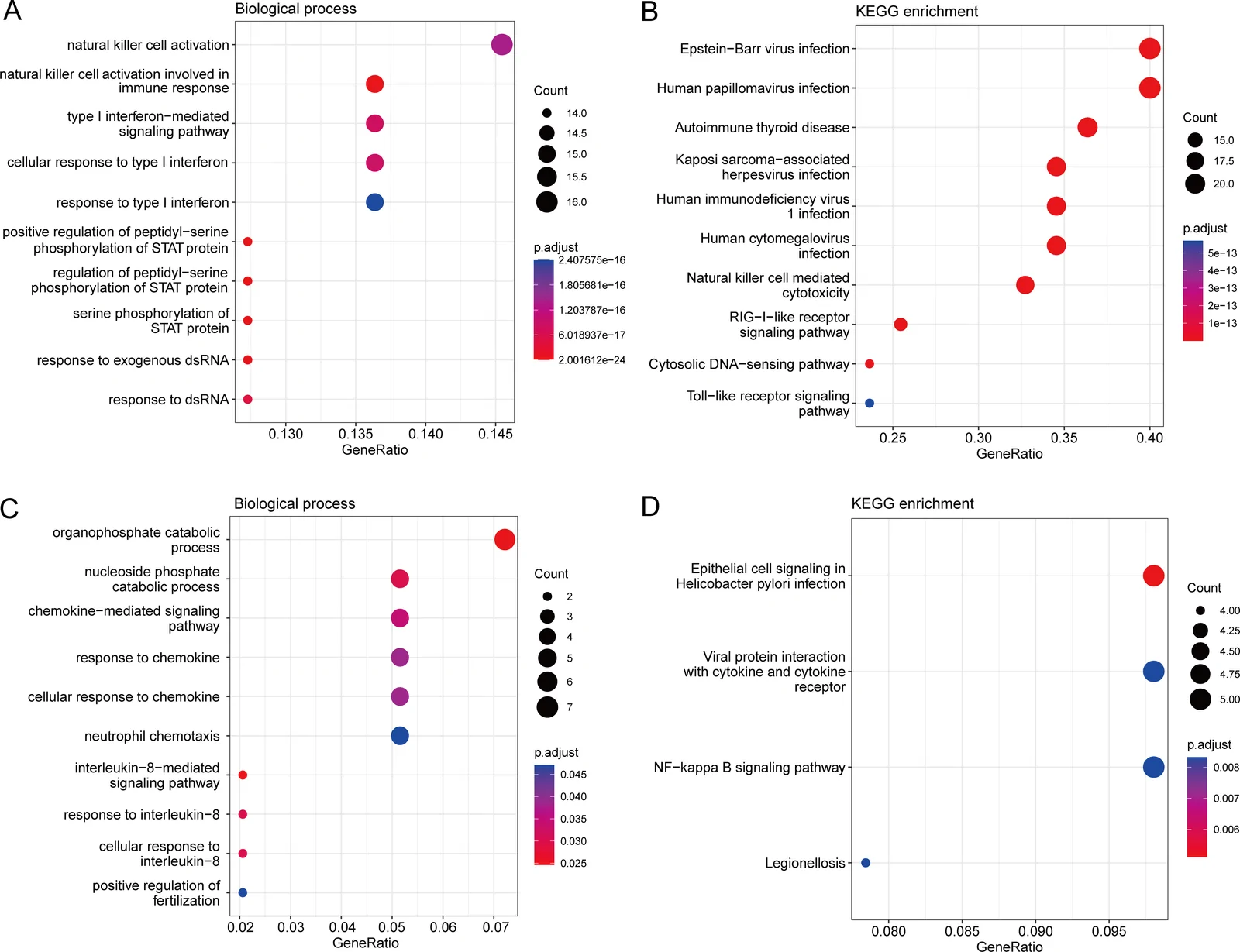
The enriched biological processes and KEGG pathways in the representative human proteins of the top 100 protein groups in the viral family of *Herpesviridae* (**A and B**) and *Filoviridae* (**C and D**).

Then, we analyzed the function of the representative human proteins from the top 100 protein groups in 11 viral families that had a median PSCC of 0.14 or larger and that contained five or more viruses used in the study (Fig. 3B). Significant enrichment of GO terms or KEGG pathways was observed in eight viral families including *Herpesviridae*, *Filoviridae*, *Arenaviridae*, *Hantaviridae*, *Nairoviridae*, *Peribunyaviridae*, *Phenuiviridae, and Retroviridae* (Fig. 5; Fig. S2). Except for the viral families of *Herpesviridae* and *Filoviridae*, only a few enriched GO terms or KEGG pathways were observed for these viral families. For the viral family of *Herpesviridae*, the enriched biological processes were mainly related to natural immunity, such as “natural killer cell activation” and “cellular response to type I interferon” (Fig. 5A); the enriched KEGG pathways were mainly related to viral infections, such as “Epstein–Barr virus infection” and “human papillomavirus infection,” and natural immunity such as “RIG-I-like receptor signaling pathway” (Fig. 5B). For the viral family of *Filoviridae*, the enriched biological processes were mainly related to response to cytokine such as interleukin (Fig. 5C); the enriched KEGG pathways were mainly related to pathogen infection such as “viral protein interaction with cytokine and cytokine receptor” (Fig. 5D).

### Prediction of mammalian virus cross-species transmission based on host proteins

**Fig 6:**
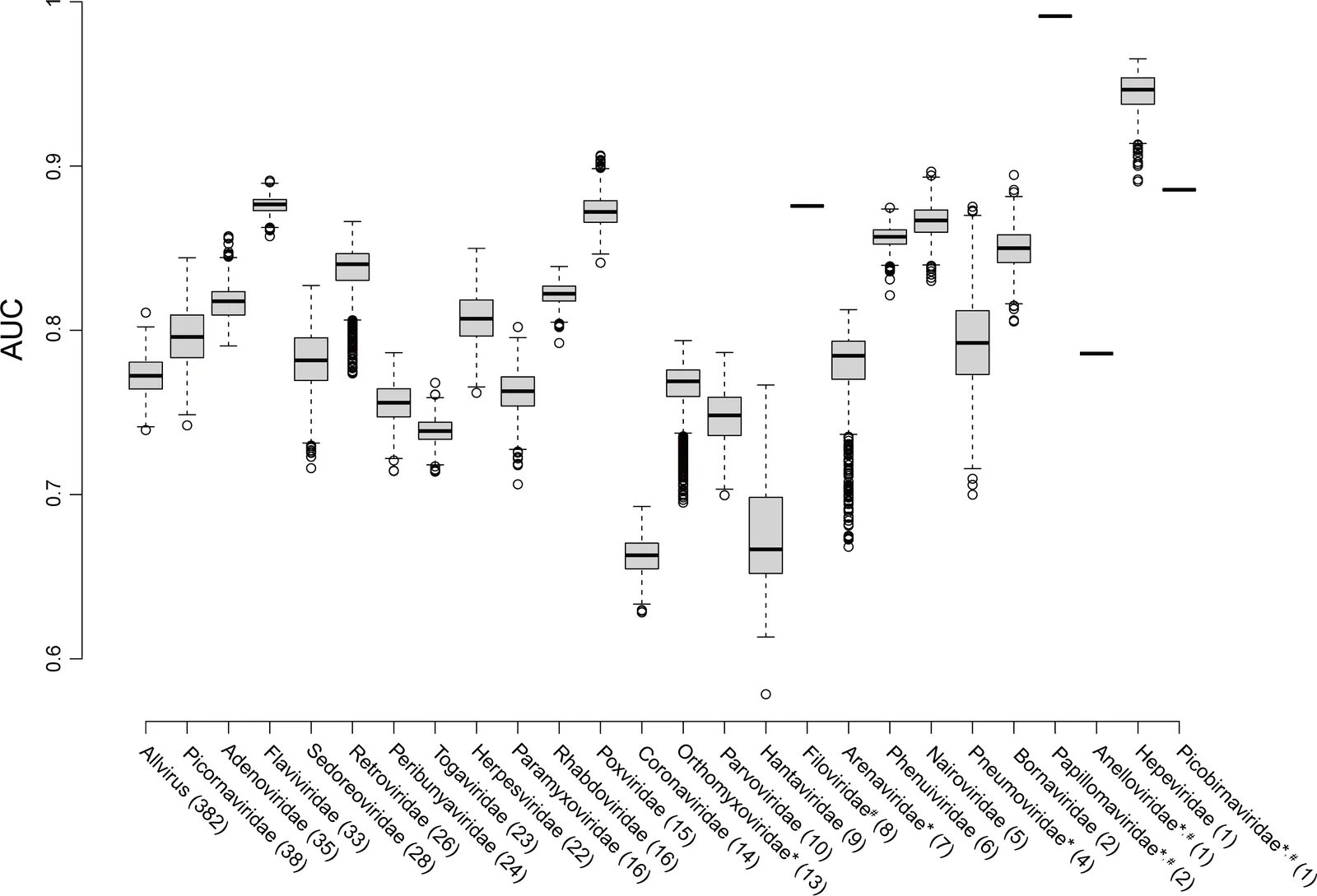
The prediction performances of RF models in predicting viral cross-species transmission based on the top 100 protein groups that correlated most to virus cross-species transmission. The leave-one-out test by mammal species was used to evaluate the model (see Materials and Methods). The boxplots shown in the figure refer to the distribution of AUCs in 1,000 times of modeling (see Materials and Methods). Viruses of the viral family *Smacoviridae* only infected two mammal species, and the leave-one-out test cannot be carried out in these viral families as there were no positive samples in the training data. “*” indicates the viral families with less than 100 protein groups that had significant correlations with virus cross-species transmission. “#” indicates viral families that had less than three mammal species in each order, and no random sampling was conducted.

The mammalian virus cross-species transmission was predicted using the RF model with the top 100 protein groups (see Materials and Methods). When considering all viruses together, the RF model performed moderately with a median AUC of 0.772 (Fig. 6). When predicting the virus cross-species transmission by viral family, the prediction performances significantly improved for most viral families. In 13 viral families, the AUC of the RF model exceeded 0.8. The viral families of *Papillomaviridae*, *Hepeviridae,* and *Picobirnaviridae* had the largest AUCs, although only one or two viruses were used in these viral families. The RF model also performed well in several viral families with more than 10 viruses, such as *Flaviviridae*, *Retroviridae,* and *Poxviridae*.

### Prediction of mammalian virus cross-species transmission based on the similarity of virus receptors

**Fig 7:**
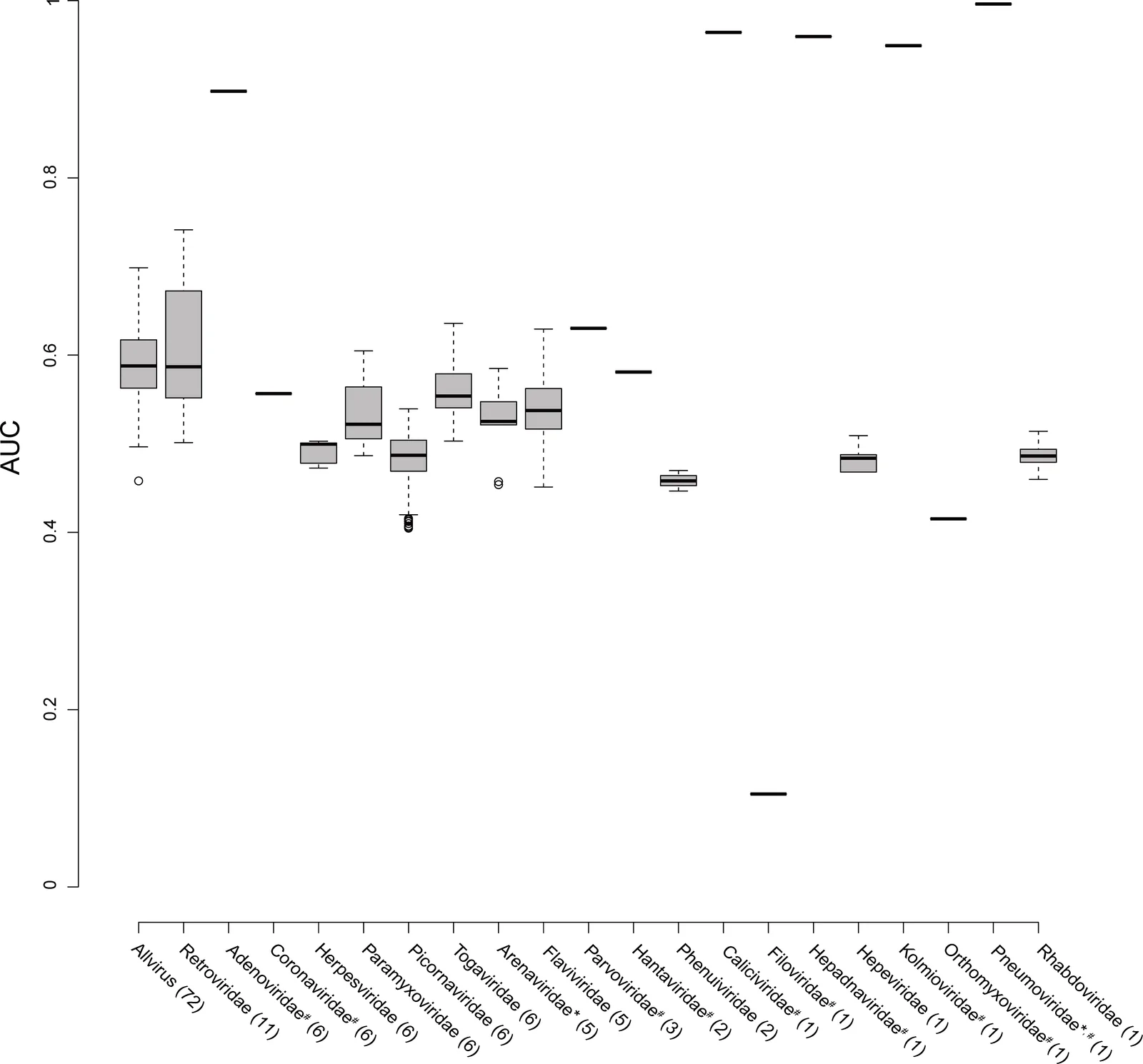
The prediction performance of the RF model in predicting mammalian virus cross-species transmission based on the protein sequence similarity between virus receptors. The leave-one-out test was used to evaluate the model (see Materials and Methods). The boxplots shown in the figure refer to the distribution of AUCs in 1,000 times of modeling (see Materials and Methods). The numbers in parentheses refer to the number of viruses in the viral family used in the study. “*” indicates the viral families with less than 100 protein groups that had significant correlations with virus cross-species transmission. “#” indicates viral families that had less than three mammal species in each order, and no random sampling was conducted.

Virus receptors have been recognized as crucial factors in virus cross-species transmission. It is hypothesized that the more similar the virus receptors of two species, the more likely the virus crosses species between the two species. Accordingly, the protein sequence similarity between virus receptors of different mammals was used to predict the potential for mammalian virus cross-species transmission (see Materials and Methods). When considering all viruses together, the RF model based on the virus receptor similarity only achieved an AUC of 0.588 (Fig. 7). When analyzing the role of the virus receptor by viral family, the AUCs of RF models decreased to about 0.5 in most viral families, indicating that the virus receptor had limited ability to predict virus cross-species transmission in these families. Interestingly, for some families such as *Hepadnaviridae* and *Kolmioviridae*, the RF models had AUCs greater than 0.90, signifying the critical role of the virus receptor in virus cross-species transmission within these viral families.

## DISCUSSION

Newly emerging viruses pose serious threats to public health and have become a global concern in recent years. To effectively prevent and control newly emerging viruses, it is crucial to identify factors associated with virus cross-species transmission and develop computational methods for predicting virus cross-species transmission. Previous studies have reported several host factors associated with virus cross-species transmission (17 – 19). In this study, numerous host proteins were found to potentially contribute to virus cross-species transmission. However, it was observed that no single host protein can solely determine the virus cross-species transmission, implying that this process is determined by multiple proteins. Viral infections are known to depend on numerous host proteins, playing critical roles in various stages of the viral life cycle, including entry into host cells, fighting against host immunity, transcription, translation, assembly, and budding. Therefore, it is likely that the cross-species transmission of mammalian viruses is influenced by the collective actions of multiple host proteins.

The study revealed that the host proteins associated with cross-species transmission of mammalian viruses were specific to viral families, with only a few overlapping proteins identified in different viral families. This finding indicates that the interactions between viruses and hosts are likely to be specific to viral families. Previous studies showed that the protein-protein interactions between viruses and hosts are virus-specific and even strain-specific (20, 34), as the virus-host interactions could be used to classify high- and low-risk human papillomaviruses (HPVs) (34). Although there are some common host proteins that interact with multiple viruses, most host proteins are only specific to one or a few viruses. Therefore, it is highly probable that the host proteins associated with cross-species transmission are specific to individual viruses, and virus-specific modeling may be a suitable approach, as supported by the observation that models focusing on viral families with one or two viruses demonstrated better performance compared to those with multiple viruses (Fig. 6).

The virus receptor has been known to play a significant role in determining the susceptibility of hosts to viral infections. A previous study conducted by Cho et al. investigated the prediction of viral cross-species transmission infection propensities based on receptor similarity using 18 virus receptors (16). They found that virus receptors have a substantial impact on host susceptibility to viral cross-species infections. This study systematically investigated the influence of the virus receptor on cross-species transmission of mammalian viruses with much larger and more diverse virus receptor proteins. Unexpectedly, the viral receptor was found to have only a trivial effect in determining the cross-species transmission of mammalian viruses. Even when analyzed by viral family, the viral receptor still had minor contributions to cross-species transmission in most viral families. This could be attributed to data bias and incompleteness, as the study only used virus receptors from 72 viruses. Additionally, the method for calculating the similarity between receptor proteins may require improvements, as only a small area of receptors interacts with the viral receptor-binding protein.

Understanding the interaction network between viruses and hosts may help much in clarifying the mechanism of virus cross-species transmission and improving the prediction model of predicting virus cross-species transmission (35, 36). Despite extensive efforts to uncover virus-host protein-protein interactions, the current knowledge remains incomplete (20, 22, 34). For example, the HVIDB, which is an up-to-date database of virus-human protein-protein interactions, contains less than 100,000 entries. Reliable computational methods are required to systematically identify high-confidence protein-protein interactions between viruses and hosts (20). While several methods for predicting virus-host protein-protein interactions have been developed in recent years, substantial improvements are still necessary to enhance their practical applications (22, 34, 37).

There were several limitations to this study. First, the interactions between mammals and viruses may be incomplete and biased. Most interactions are biased toward the *Primates* and *Artiodactyla* because they have been more extensively investigated. Additionally, the absence of viruses in a species may be due to sampling bias, as it is challenging to determine definitively whether a virus did not infect a particular species. Further data and research are required to validate and refine the findings. Second, the models of predicting the cross-species transmission of mammalian viruses showed poor performances in some viral families, indicating that some factors contributing to the virus cross-species transmission may have been overlooked in these models. Integrating both macro-level and molecular-level factors could potentially enhance the predictive models for cross-species transmission of mammalian viruses.

Overall, this is the first study to systematically investigate the host proteins associated with cross-species transmission of mammalian viruses and predict the cross-species transmission of the mammalian virus based on both virus receptors and other host proteins. It deepens our understanding of the mechanism of virus cross-species transmission and provides a framework for predicting the cross-species transmission of mammalian viruses.

## Data Availability

All data used in the study are available in the supplementary materials.

## SUPPLEMENTAL MATERIAL

Fig. S1 spectrum.05368-22-s0001.docx The number of species in orthogroup.

Fig. S2 spectrum.05368-22-s0002.docx The enriched GO terms and KEGG pathways in eight viral families.

Table. S1 spectrum.05368-22-s0003.xlsx The interactions between mammalian viruses and mammals.

Table. S2 spectrum.05368-22-s0004.xlsx Virus receptor used in the study.

Table. S3 spectrum.05368-22-s0005.xlsx The top 100 protein groups in each viral family.

Table. S4 spectrum.05368-22-s0006.docx The number of positive and negative samples based on host proteins.

Table. S5 spectrum.05368-22-s0007.docx The number of positive and negative samples based on similarity of virus receptors.

ASM does not own the copyrights to Supplemental Material that may be linked to, or accessed through, an article. The authors have granted ASM a non-exclusive, world-wide license to publish the Supplemental Material files. Please contact the corresponding author directly for reuse.
