# Supplementary material for: Prediction of mammalian virus cross-species transmission based on host proteins
Source: Microbiol Spectr. 2023 Sep 27;11(5):e05368-22. doi: 10.1128/spectrum.05368-22 (PMC10581197; doi:10.1128/spectrum.05368-22)

**Figure S1**. The accumulated number of orthogroups which appeared in a given number of species in orthogroup.


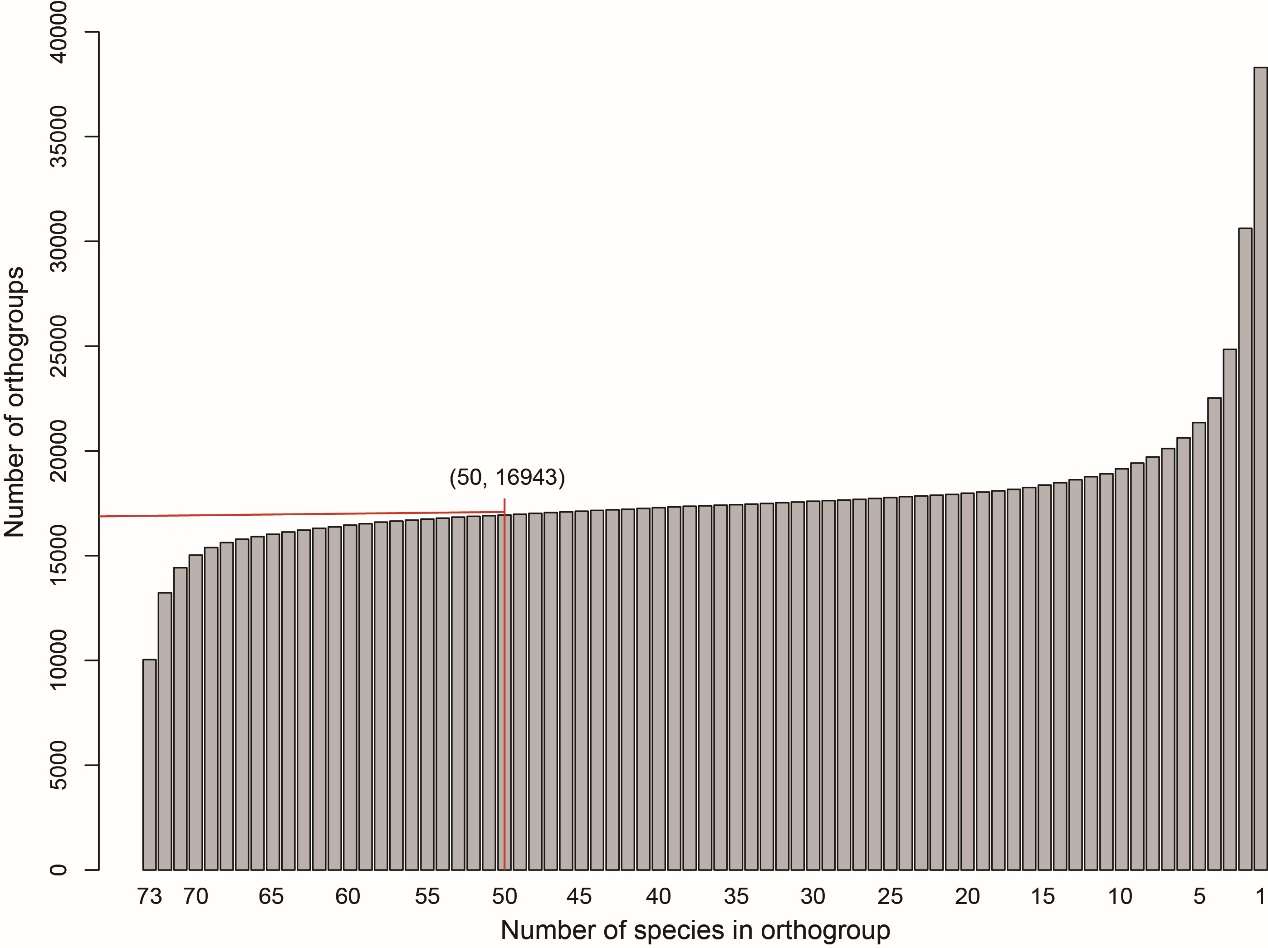

Supplement: Fig. S1 — The number of species in orthogroup. [file spectrum.05368-22-s0001.docx]
