# Supplementary material for: Prediction of mammalian virus cross-species transmission based on host proteins
Source: Microbiol Spectr. 2023 Sep 27;11(5):e05368-22. doi: 10.1128/spectrum.05368-22 (PMC10581197; doi:10.1128/spectrum.05368-22)
Supplement: Fig. S2 — The enriched GO terms and KEGG pathways in eight viral families. [file spectrum.05368-22-s0002.docx]

**Figure S2.** The enriched GO terms and KEGG pathways in eight viral families including *Herpesviridae*, *Filoviridae*, *Arenaviridae*, *Hantaviridae*, *Nairoviridae*, *Peribunyaviridae*, *Phenuiviridae and Retroviridae.*

**Arenaviridae**


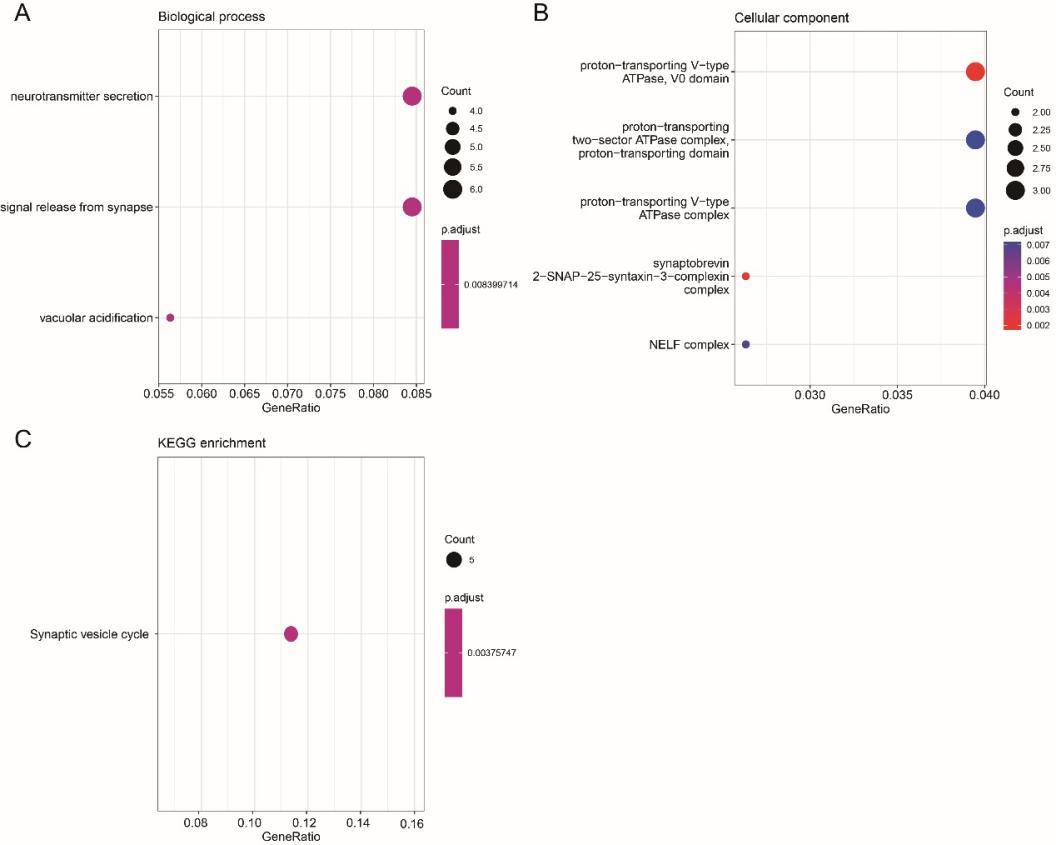


**Filoviridae**

**
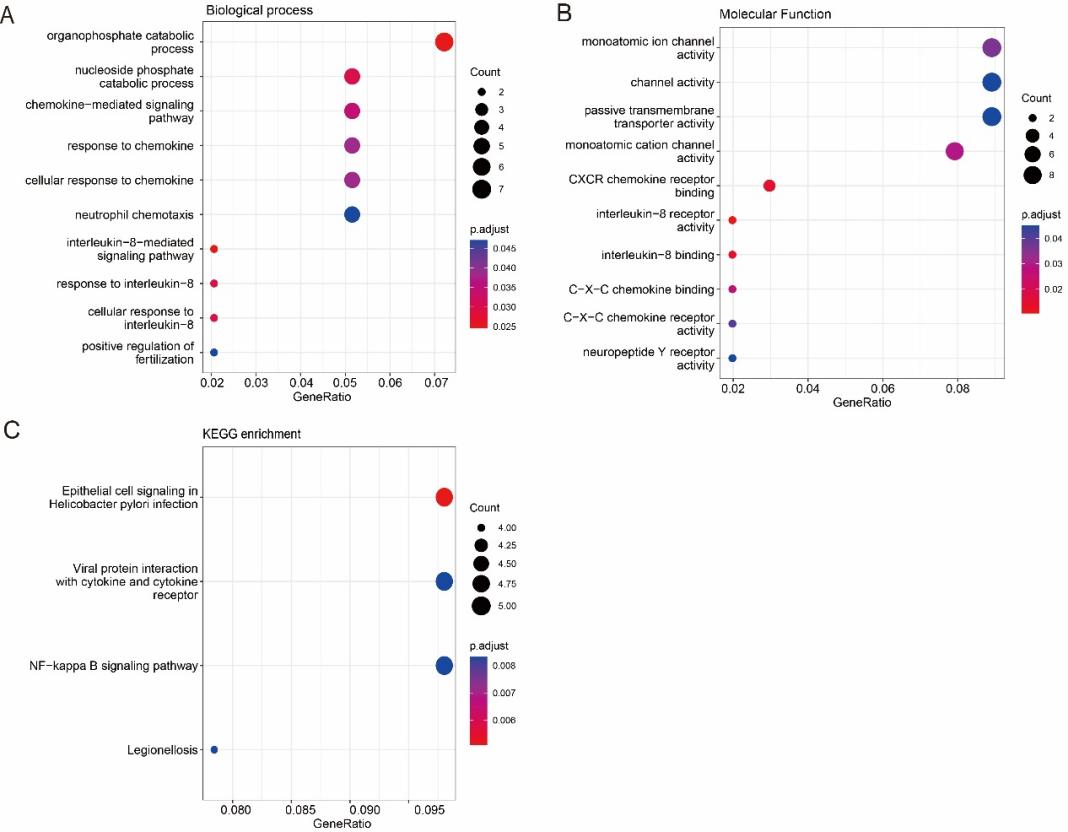
**

**Hantaviridae**


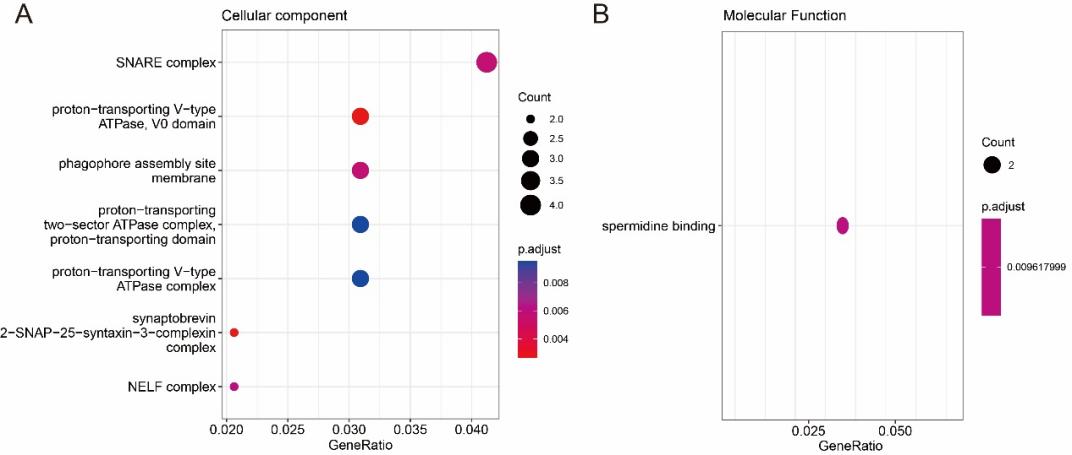


**Herpesviridae**


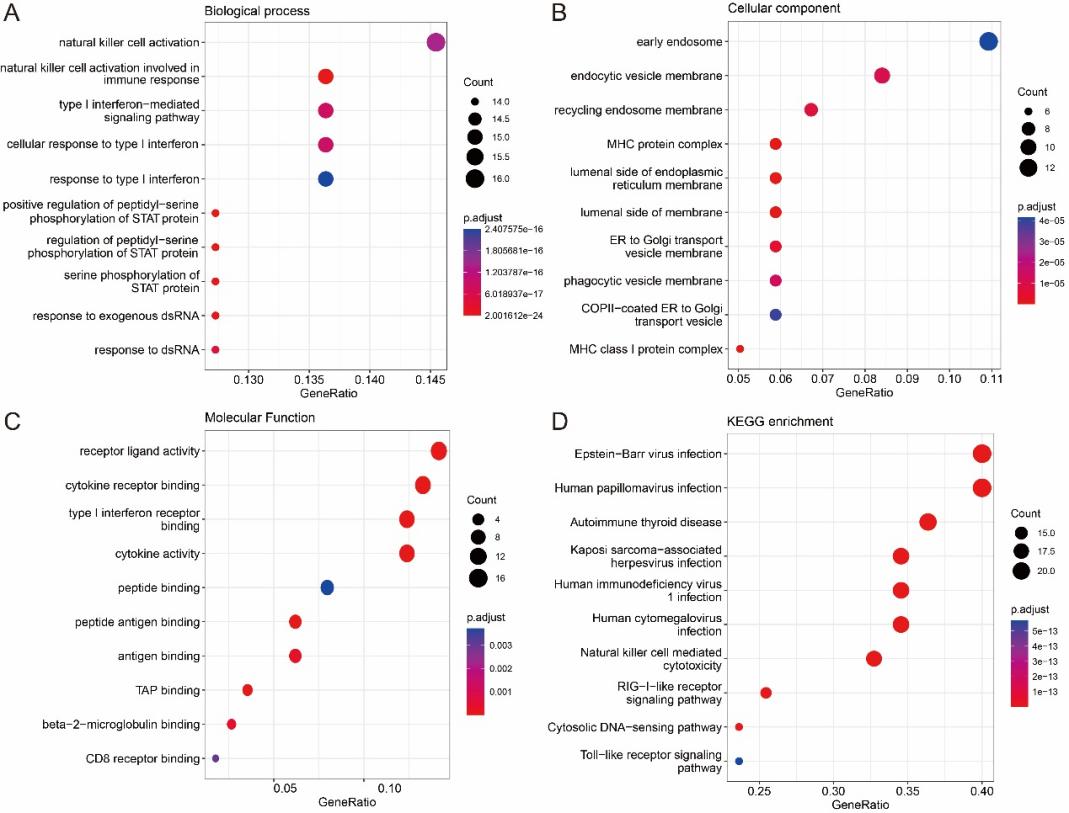


**Nairoviridae**


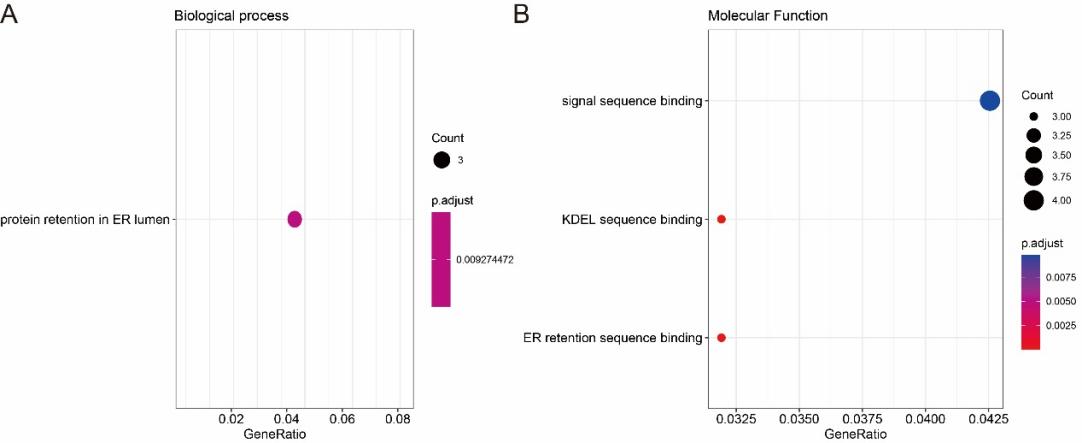


**Peribunyaviridae**

**
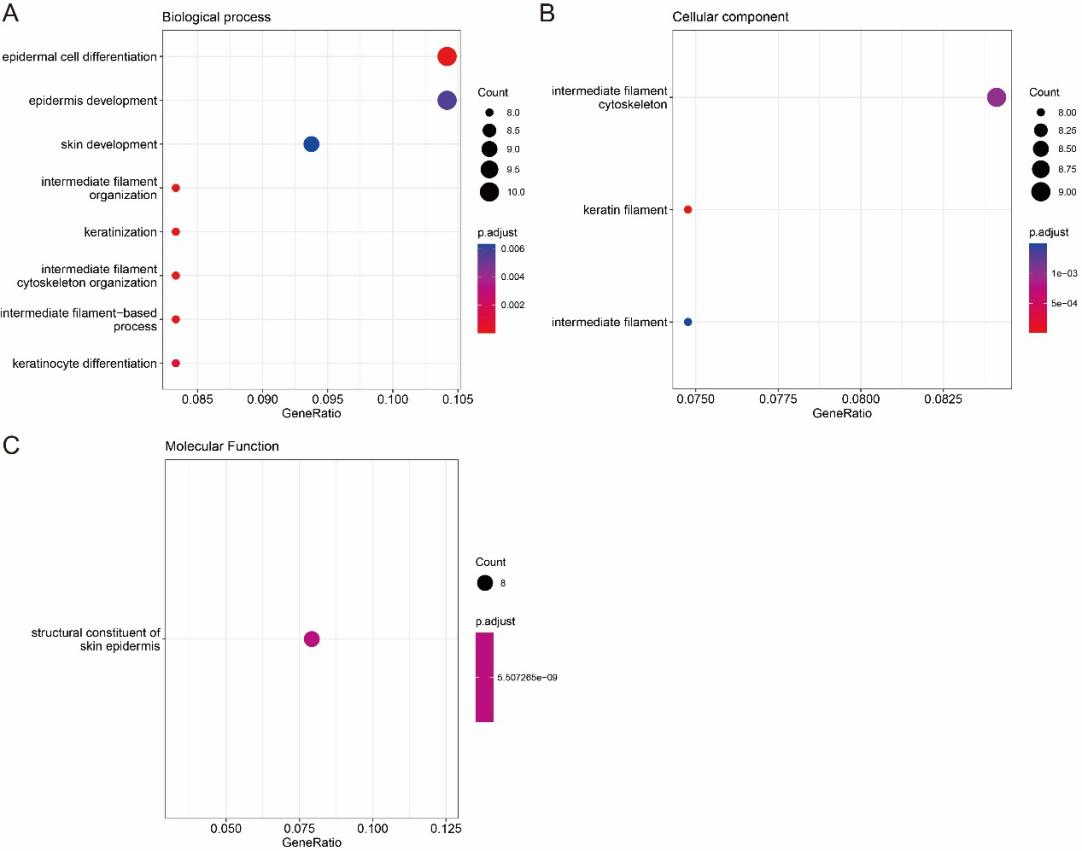
**

**Phenuiviridae**

**
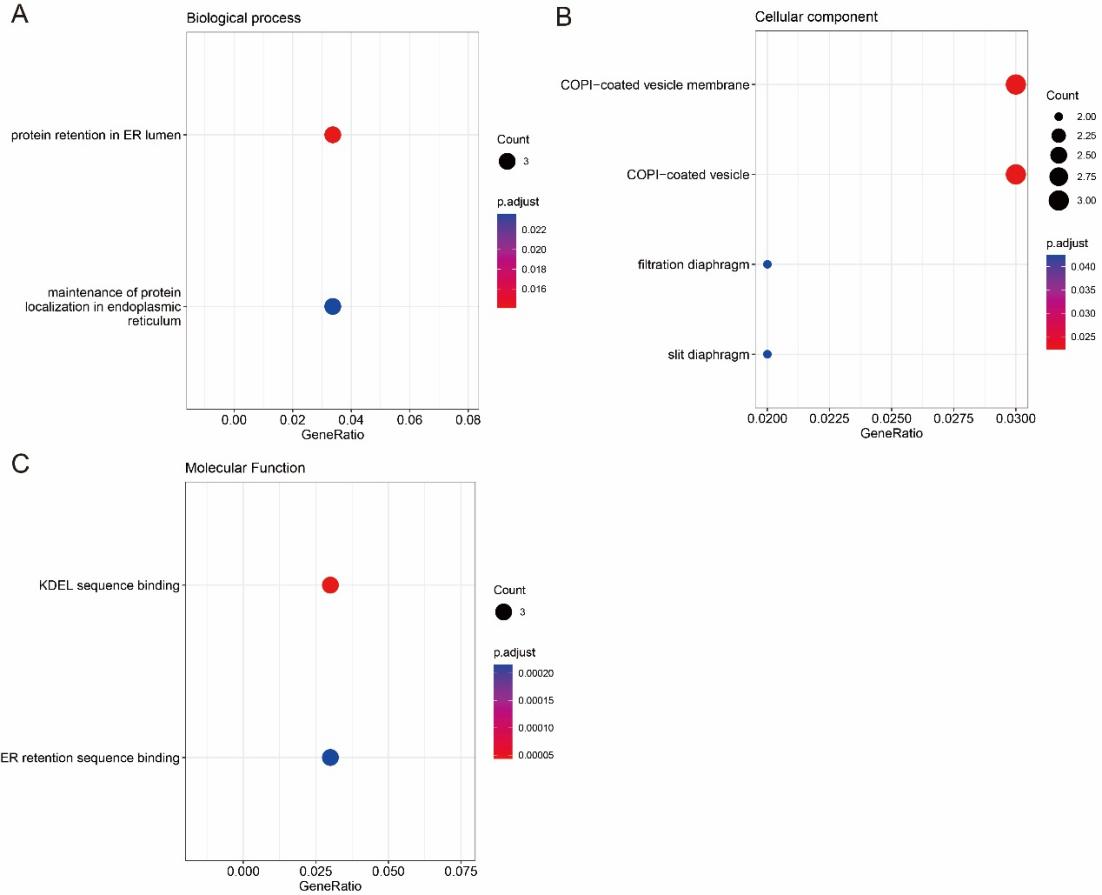
**

**Retroviridae**

**
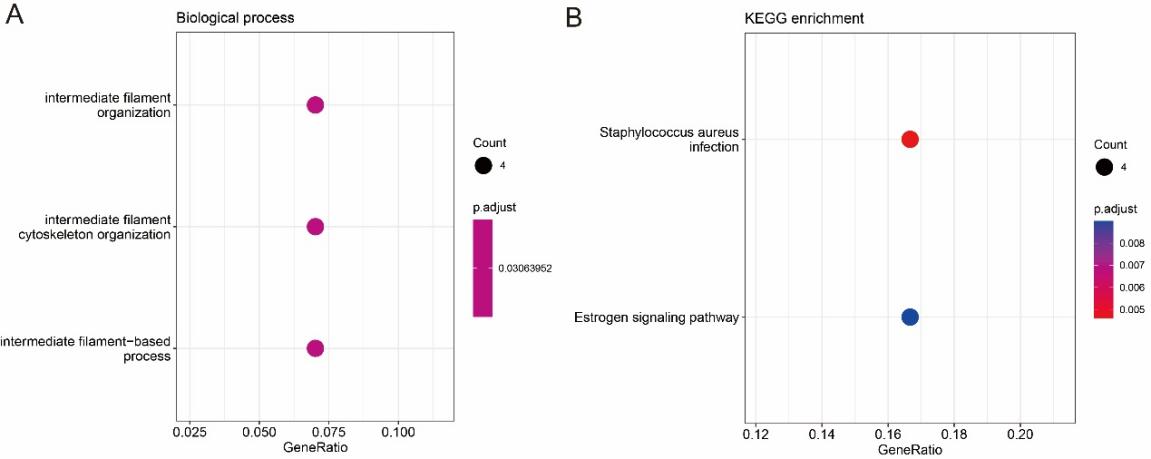
**
