# Supplementary material for: Prediction of mammalian virus cross-species transmission based on host proteins
Source: Microbiol Spectr. 2023 Sep 27;11(5):e05368-22. doi: 10.1128/spectrum.05368-22 (PMC10581197; doi:10.1128/spectrum.05368-22)
Supplement: Table. S4 — The number of positive and negative samples based on host proteins. [file spectrum.05368-22-s0006.docx]

**Table S4.** The number of positive and negative samples used in prediction of mammalian virus cross-species transmission based on host proteins.

| **Viral family** | **Virus num** | **Positive num** | **Negative num** | **Viral family** | **Virus num** | **Positive num** | **Negative num** |
| --- | --- | --- | --- | --- | --- | --- | --- |
| All virus | 382 | 2113 | 15283 | Papillomaviridae | 2 | 7 | 342 |
| Adenoviridae | 35 | 58 | 2867 | Paramyxoviridae | 16 | 141 | 1859 |
| Anelloviridae | 1 | 15 | 342 | Parvoviridae | 10 | 17 | 339 |
| Arenaviridae | 7 | 21 | 1015 | Peribunyaviridae | 24 | 129 | 2981 |
| Bornaviridae | 2 | 81 | 861 | Phenuiviridae | 6 | 34 | 838 |
| Coronaviridae | 14 | 22 | 411 | Picobirnaviridae | 1 | 15 | 354 |
| Filoviridae | 8 | 14 | 1201 | Picornaviridae | 38 | 209 | 829 |
| Flaviviridae | 33 | 307 | 1031 | Pneumoviridae | 4 | 13 | 667 |
| Hantaviridae | 9 | 15 | 204 | Poxviridae | 15 | 98 | 1001 |
| Hepeviridae | 1 | 28 | 432 | Retroviridae | 26 | 96 | 412 |
| Herpesviridae | 22 | 112 | 1121 | Rhabdoviridae | 16 | 283 | 1077 |
| Nairoviridae | 5 | 70 | 522 | Sedoreoviridae | 28 | 107 | 1855 |
| Orthomyxoviridae | 12 | 30 | 864 | Togaviridae | 23 | 155 | 3242 |
