# Supplementary material for: Prediction of mammalian virus cross-species transmission based on host proteins
Source: Microbiol Spectr. 2023 Sep 27;11(5):e05368-22. doi: 10.1128/spectrum.05368-22 (PMC10581197; doi:10.1128/spectrum.05368-22)
Supplement: Table. S5 — The number of positive and negative samples based on similarity of virus receptors. [file spectrum.05368-22-s0007.docx]

**Table S5**. The number of positive and negative samples used in prediction of mammalian virus cross-species transmission based on similarity of virus receptors.

| **Viral family** | **Virus num** | **Positive num** | **Negative num** | **Viral family** | **Virus num** | **Positive num** | **Negative num** |
| --- | --- | --- | --- | --- | --- | --- | --- |
| All virus | 72 | 769 | 16230 | Kolmioviridae | 1 | 1 | 128 |
| Adenoviridae | 6 | 14 | 994 | Orthomyxoviridae | 1 | 3 | 183 |
| Arenaviridae | 5 | 12 | 793 | Paramyxoviridae | 6 | 42 | 1529 |
| Caliciviridae | 1 | 3 | 186 | Parvoviridae | 3 | 5 | 445 |
| Coronaviridae | 6 | 13 | 937 | Phenuiviridae | 2 | 29 | 573 |
| Filoviridae | 1 | 1 | 124 | Picornaviridae | 6 | 131 | 2001 |
| Flaviviridae | 5 | 112 | 1671 | Pneumoviridae | 1 | 1 | 128 |
| Hantaviridae | 2 | 4 | 312 | Retroviridae | 11 | 25 | 1694 |
| Hepadnaviridae | 1 | 3 | 189 | Rhabdoviridae | 1 | 210 | 924 |
| Hepeviridae | 1 | 28 | 464 | Togaviridae | 6 | 71 | 1618 |
| Herpesviridae | 6 | 61 | 1338 |  |  |  |  |
